# Supplementary figures and images for: Trends in antimicrobial resistance amongst Salmonella Typhi in Bangladesh: A 24-year retrospective observational study (1999–2022)
Source: PLoS Negl Trop Dis. 2024 Oct 4;18(10):e0012558. doi: 10.1371/journal.pntd.0012558 (PMC11482714; doi:10.1371/journal.pntd.0012558)

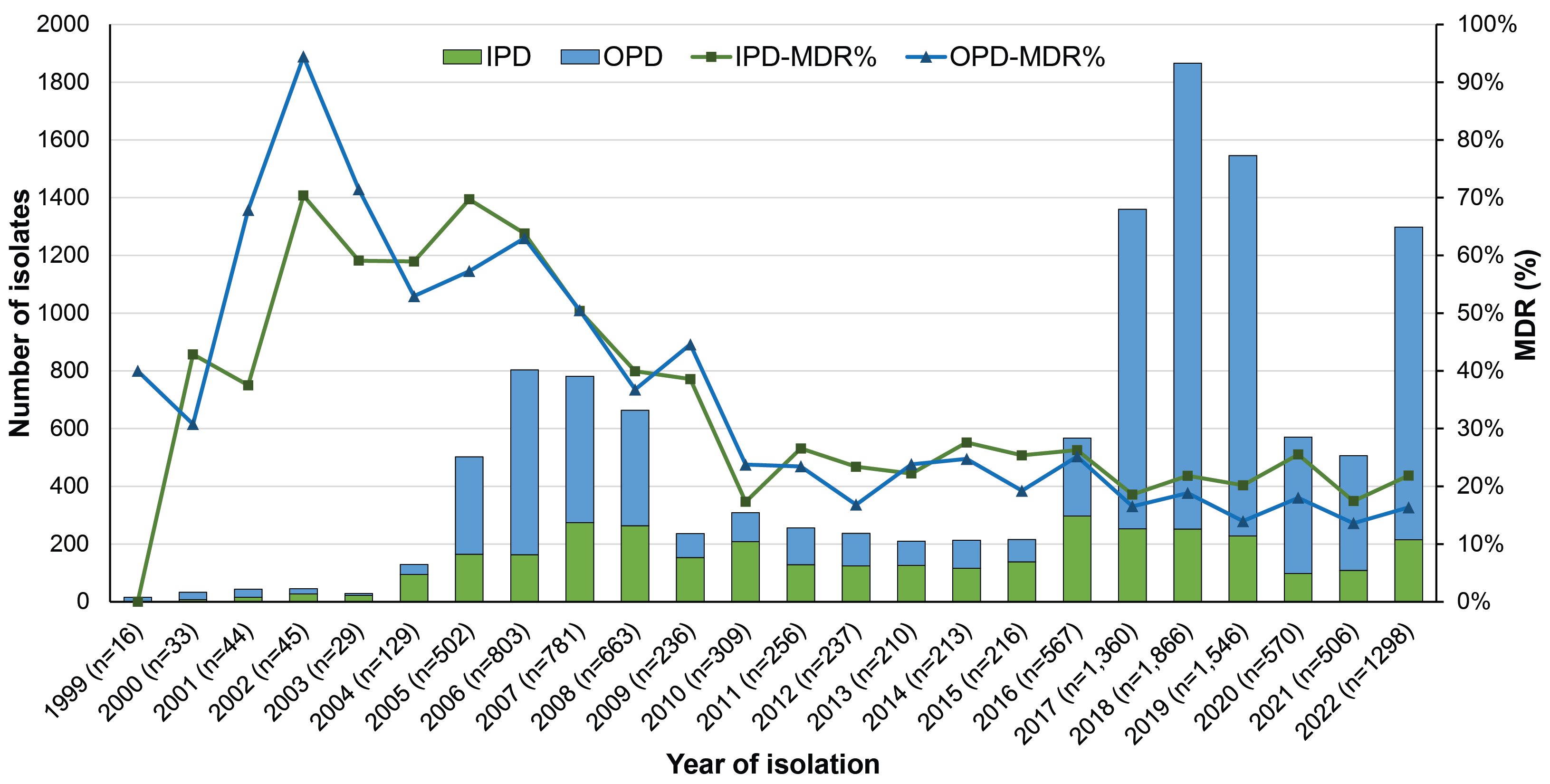

Supplement: S1 Fig — Yearly hospitalized (IPD) and outpatient (OPD) case numbers (on the left y-axis) are presented by the year of isolation. The x-axis labels indicate the number of isolates collected each year. Percentages of MDR for IPD and OPD cases per year represented as lines on the right y-axis. (TIF) [file pntd.0012558.s002.tif]
