# Supplementary material for: Trends in antimicrobial resistance amongst Salmonella Typhi in Bangladesh: A 24-year retrospective observational study (1999–2022)
Source: PLoS Negl Trop Dis. 2024 Oct 4;18(10):e0012558. doi: 10.1371/journal.pntd.0012558 (PMC11482714; doi:10.1371/journal.pntd.0012558)
Supplement: Attachment — Submitted filename: Response_to_Reviewer_Comments.docx [file pntd.0012558.s003.docx]

**Reviewer's Responses to Questions**

**Key Review Criteria Required for Acceptance? As you describe the new analyses required for acceptance, please consider the following:**

**Methods**

-Are the objectives of the study clearly articulated with a clear testable hypothesis stated?</br> -Is the study design appropriate to address the stated objectives?</br> -Is the population clearly described and appropriate for the hypothesis being tested?</br> -Is the sample size sufficient to ensure adequate power to address the hypothesis being tested?</br> -Were correct statistical analysis used to support conclusions?</br> -Are there concerns about ethical or regulatory requirements being met?</br></br>

**Reviewer #1:**

**Methods Section:**

Objectives and Hypothesis:

The objectives of the study are clearly articulated in the methodology section. The study aims to explore antimicrobial resistance trends in Salmonella Typhi isolates from Bangladesh over a 24-year period and compare them with antibiotic consumption patterns to optimize antibiotic stewardship strategies for the country. Although the methodology does not explicitly state a testable hypothesis, the objectives are sufficiently clear and specific.

Study Design Appropriateness:

The retrospective observational study design is appropriate for addressing the stated objectives. It allows for the examination of trends in antimicrobial resistance over time and comparison with antibiotic consumption data, providing valuable insights into the dynamics of antimicrobial resistance in Salmonella Typhi in Bangladesh.

Description and Appropriateness of Population:

The population under surveillance consists of individuals diagnosed with enteric fever, predominantly pediatric patients (<18 years of age), in Dhaka, Bangladesh. Given that typhoid fever predominantly affects children in this region, focusing on pediatric populations is appropriate for the hypothesis being tested.

Sample Size Adequacy:

The methodology does not provide specific details regarding sample size calculation. However, the study spans 24 years and includes over 12,000 cases of Salmonella Typhi, indicating a substantial sample size for analyzing trends in antimicrobial resistance over time. This sample size should ensure adequate power to address the study's objectives effectively.

Statistical Analysis Support:

The statistical analyses described, including Pearson's correlation coefficients, generalized additive models, and local polynomial regression, are appropriate for exploring correlations and non-linear patterns in antimicrobial resistance and consumption data. These analyses support the conclusions drawn from the study findings.

Ethical and Regulatory Considerations:

The methodology does not explicitly mention ethical or regulatory requirements. However, it can be inferred that the study adheres to ethical guidelines, as it involves retrospective analysis of anonymized data collected as part of routine surveillance activities and diagnostic procedures. Additionally, the authors stated from line 387 to 392 that it have received ethical approval from relevant institutional review boards or ethical committees. (the Ethics Review Committees (ERC) of Bangladesh Shishu Hospital and Institute (BSHI).

- We thank the reviewer for their positive words.

**Reviewer #2:**

The observational study describes Salmonella typhi antibiotic resistance in isolates from select health facilities in Dhaka, Bangladesh, over more than 20 years. The associations with use of those antibiotics in Bangladesh over the same period is explored. The objectives of the study are clearly articulated and the design appropriate to address the stated objectives. The population is clearly described. The sample size is sufficient. The statistical analysis is sound. I do not have concerns about ethical or regulatory requirements being unmet.

- We appreciate the reviewer for their positive comments.

**Reviewer #3:**

-Are the objectives of the study clearly articulated with a clear testable hypothesis stated? **Yes** -Is the study design appropriate to address the stated objectives? **Yes** -Is the population clearly described and appropriate for the hypothesis being tested? **Yes** -Is the sample size sufficient to ensure adequate power to address the hypothesis being tested? **Yes** -Were correct statistical analysis used to support conclusions? **Yes** -Are there concerns about ethical or regulatory requirements being met? **Yes**

- We value the reviewer’s positive comments and appreciate their support.

**Reviewer #4:**

Thank you for the opportunity to review this manuscript.

Very clear methods; clear study design.

Appropriate statistical methods.

- We thank the reviewer for their favorable feedback.

**Reviewer #5:**

The methods are clear and well-written.

- We appreciate the reviewer for their positive comment.

Minor comments:

Line 162 & 177: Please remove dash for "co-trimoxazole"

- We revised the term accordingly (Line 165 and 180 in the revised manuscript).

--------------------

**Results**

-Does the analysis presented match the analysis plan?</br> -Are the results clearly and completely presented?</br> -Are the figures (Tables, Images) of sufficient quality for clarity?</br></br>

**Reviewer #1:**

**Results:**

Matching Analysis Plan:

The analysis presented in the results section align with the analysis plan outlined in the methodology. The results address the key objectives of the study, including trends in antimicrobial resistance among Salmonella Typhi isolates, changes in antibiotic consumption patterns, and correlations between antimicrobial resistance and antibiotic consumption. The statistical methods described in the methodology, such as Pearson's correlation coefficients and generalized additive models, are appropriately utilized to analyze the data and support the conclusions drawn.

Clarity and Completeness of Presentation:

The results are generally clear and comprehensive, providing detailed findings on antimicrobial resistance trends, antibiotic consumption patterns, and correlations between the two. The text effectively summarizes the main observations and trends identified during the 24-year surveillance period.

Quality of Figures (Tables, Images):

The figures, tables, and images presented in the results section contribute to the clarity and understanding of the findings. They are adequately labeled and described, allowing readers to interpret the data accurately.

- We appreciate the reviewer for their positive comments.

**Reviewer #2:**

The results are clear and well presented in the figures.

- We thank the reviewer for their favorable feedback.

**Reviewer #3:**

-Does the analysis presented match the analysis plan? **Yes** -Are the results clearly and completely presented? **Yes** -Are the figures (Tables, Images) of sufficient quality for clarity? **Yes**

- We value the reviewer’s positive comments.

**Reviewer #4:**

Tables and images are helpful.

Results are clear, and presented well.

- We thank the reviewer for their favorable feedback.

**Reviewer #5:**

The MIC50 of ceftriaxone and ciprofloxacin was calculated in the method section, but the MIC50 of ciprofloxacin can not be found in the results.

- Thanks to the reviewer for the comment. The MIC50 for ciprofloxacin is now included in Line 222-225.

Line 222-225: “The MIC trend for ciprofloxacin remained stable at approximately 0.25 mg/L throughout the entire study period (Fig 3A). The yearly MIC50 also remained unchanged since 2004 at 0.25 mg/L, except in 2012 (MIC_50_ = 0.125 mg/L).”

Minor comments:

Line 208: Please remove full form of MIC

- Thanks to the reviewer. We made the changes accordingly (Line 219 of the revised manuscript).

--------------------

**Conclusions**

-Are the conclusions supported by the data presented?</br> -Are the limitations of analysis clearly described?</br> -Do the authors discuss how these data can be helpful to advance our understanding of the topic under study?</br> -Is public health relevance addressed?</br></br>

**Reviewer #1:**

**Conclusion:**

Conclusions Supported by Data Presented:

The conclusions drawn in the study are well-supported by the data presented. The analysis of antimicrobial resistance trends among Salmonella Typhi isolates over a 24-year period, alongside antibiotic consumption patterns, provides a robust basis for the conclusions regarding the declining trends in multidrug resistance and the potential efficacy of reintroducing first-line antimicrobials for typhoid fever treatment in Bangladesh.

Clear Description of Limitations:

The study's limitations are adequately described, including potential biases associated with physician discretion in selecting cases for blood cultures, the retrospective nature of the study design, and any methodological constraints related to data collection and analysis. This transparency enhances the credibility of the findings and ensures that readers can interpret the results within the appropriate context.

Discussion on Advancing Understanding:

The authors discuss how the data presented in the study can advance our understanding of the topic under study. Specifically, they highlight the significance of the findings as a baseline for monitoring the impact of future interventions, such as typhoid conjugate vaccines, on typhoid burden and associated antimicrobial resistance. This forward-looking perspective underscores the broader implications of the research beyond its immediate findings.

Addressing Public Health Relevance:

Public health relevance is effectively addressed throughout the study, particularly in the discussion of the implications of the findings for antibiotic stewardship strategies and the management of typhoid fever in Bangladesh. By highlighting the declining trends in multidrug resistance and the potential reintroduction of first-line antimicrobials, the study directly addresses public health concerns related to antimicrobial resistance and infectious disease management.

- We thank the reviewer for their positive feedback.

**Reviewer #2:**

The conclusions are appropriate, the important limitations are discussed, and the potential utility of the results for public health, is discussed. I have suggested enriched discussion of aspects in 'summary and general comments'.

- We thank the reviewer for their comments. We addressed the suggestions in the discussion according to reviewer’s general comments (details are added with the comments).

**Reviewer #3:**

-Are the conclusions supported by the data presented? **Yes** -Are the limitations of analysis clearly described? **Yes** -Do the authors discuss how these data can be helpful to advance our understanding of the topic under study? **Yes** -Is public health relevance addressed? **Yes**

- We thank the reviewer for their positive feedback.

**Reviewer #4:**

More on limitations would be beneficial, particularly discussing the differences.

- We have revised the limitations paragraph in the Discussion and added additional lines regarding the sampling strategy, MIC methods, and the lack of consumption data for amoxicillin, chloramphenicol, and ceftriaxone. This is included in line 360-378.

Line 360-378: “The results of our study should be considered within the context of several limitations. First, the two hospitals participating in the surveillance were among the largest pediatric reference hospitals in Bangladesh, resulting in a bias towards younger patients. Second, all study sites were in Dhaka, which may not represent other parts of Bangladesh. However, previous data reported 57% seropositivity for typhoid fever in Dhaka, reflecting the high number of cases in this city [6]. Third, many typhoid patients in Bangladesh are empirically treated without blood culture confirmation. No community-based enteric fever surveillance accounts for this, limiting comparisons. Fourth, the sampling strategy of our study did not include a calculated sample size, as it was conducted as part of an ongoing laboratory surveillance for enteric fever. Additionally, blood cultures were suggested by treating physicians, which may have introduced biases. Fifth, our broth microdilution plates were designed to determine MICs up to a maximum of 256 mg/L, and it prevented us from accurately measuring the exact ceftriaxone MICs of the two resistant isolates, which were excluded from subsequent analysis. Finally, national antibiotic consumption data from IQVIA-MIDAS® database were used in this study, as regional or disease-specific data were unavailable. Since pharmaceutical sales data is proprietary information, we could not find any other source providing yearly comprehensive sales data of all antimicrobials available in Bangladesh. Consequently, we could not validate these data points with other sources. Additionally, antibiotic consumption data for amoxicillin, chloramphenicol, and ceftriaxone were unavailable.”

**Reviewer #5:**

The correlation between the reduction in % AMR against first-line drugs and the decrease in antibiotic consumption is evident. However, the proposition of "reintroducing first-line antimicrobials for treating typhoid fever " (Line 363) warrants careful consideration due to the potential for escalating MDR isolates. In other bacterial infections, adherence to WHO guidelines is crucial, recommending the discontinuation of treatment regimens if failure rates exceed 5% or if resistance is detected in 5% or more of tested isolates (source: <https://www.who.int/data/gho/indicator-metadata-registry/imr-details/5550>).

- We appreciate the valuable comment from the reviewer regarding the reintroduction of first-line antimicrobials for treating typhoid fever.

We acknowledge the concerns about the potential escalation of multidrug-resistant (MDR) isolates with the reintroduction of first-line drugs. Our discussion underscores several points relevant to this issue. Our data shows a significant decline in resistance patterns for first-line antimicrobials (amoxicillin, chloramphenicol, and cotrimoxazole), correlating with a decrease in MDR typhoid cases. Since 2017, only 17% (15%–19%, 95% CI) of typhoid cases have remained resistant to these first-line drugs (Line 295-297).

We completely agree that the proposition of reintroducing these drugs must be approached with caution. Continuous clinical, laboratory, and genomic surveillance is essential to monitor any changes in resistance patterns. Once the typhoid conjugate vaccine is introduced, the expected reduction in typhoid cases will enable more focused antimicrobial stewardship strategies, which are crucial in preventing the recurrence of MDR typhoid. We have made this more explicit in lines 353-358 based on the reviewer’s comments.

Line 353-358: “Due to these genomic changes, the re-emergence of MDR would require the frequency of H58 lineage to fall below 5% before reintroduction of older drugs. Continuous clinical, laboratory, and genomic surveillance in the post-TCV era will be essential to secure a safe window for the use of the old antimicrobials [43]. Monitoring antimicrobial use and resistance remains crucial for successfully treating typhoid and other infections effectively.”

Minor comments:

Line 361: Please remove full form of AMR.

- Thanks to the reviewer for the correction. We made the changes accordingly (Line 380 in the revised manuscript).

--------------------

**Editorial and Data Presentation Modifications?**

Use this section for editorial suggestions as well as relatively minor modifications of existing data that would enhance clarity. If the only modifications needed are minor and/or editorial, you may wish to recommend “Minor Revision” or “Accept”.

**Reviewer #1:**

Accept with minor revisions, including clarification on sample size calculation and explicit mention of ethical considerations in the methods section.

- Thanks to the reviewer for their valuable feedback and positive comments on our manuscript. Regarding the comment on sample size calculation, we would like to clarify that our study did not include a sample size calculation. The data presented in our study is part of an ongoing surveillance started in 1999 and across multiple hospital sites since 2004. Therefore, the study data represents all typhoid-positive cases found from the surveillance. We have included this in the limitations section (Line 367-370).

Line 367-370: “Fourth, the sampling strategy of our study did not include a calculated sample size, as it was conducted as part of an ongoing laboratory surveillance for enteric fever. Additionally, blood cultures were suggested by treating physicians, which may have introduced biases.”

- Regarding the ethical considerations, we have added an ethical statement at the end of the Method section (Line 189-195).

Line 189-195: “All study protocols received approval from the Ethics Review Committees (ERC) of Bangladesh Shishu Hospital and Institute (BSHI) at Dhaka, Bangladesh. Informed written consent and clinical information were obtained from parents/legal guardians for hospitalized cases. For out-patient cases, no formal consent was obtained as blood samples were collected as part of routine clinical care at the discretion of the treating physician and data from routine clinical care were retrospectively included without identifiable information.”

**Reviewer #2:** -

**Reviewer #3:** I would like to suggest a few minor edits for improvement.

1. In Fig 3., I would suggest taking a logarithm of 2 on the y-axis to accurately reflect the effect of doubling dilution in minimum inhibitory concentration. Without the logarithm, the distance between MICs, 4 and 8 should not be same as the distance between MICs, 0.25 and 0.5.

- Thanks to the reviewer for the insightful suggestion regarding the y-axis of Figure 3.

Figure 3 is based on a logarithm of 2 scale on the y-axis. For instance, 2^0^ is 1, and 2^-1^ is 0.5, and so on. To simplify the interpretation for readers, we have presented the MIC values directly on the y-axis. Using values such as 2^-1^ instead of 0.5 might be more complex for readers to understand. We have included an explanation of this approach in the figure legends (Line 238-239 and 590-591).

Line 238-239 and 590-591: “The y-axis is drawn based on a logarithm of 2 scale; MIC values are shown directly (for example, 0.5 instead of 2^-1^).”

2. How could you prevent MDR from recurring after you reintroduce first-line drugs?

- Thanks to the reviewer for the thoughtful question regarding the prevention of MDR typhoid recurrence after reintroducing first-line drugs (amoxicillin, chloramphenicol, and cotrimoxazole).

We acknowledge that there is a potential risk of MDR recurrence involved with increased use of these older drugs. Recent genomic studies indicate that MDR genes in initial H58 *Salmonella* Typhi isolates were carried on an IncHI1 plasmid, but newer isolates have integrated these genes into a chromosomal island, reducing the plasmid-imposed fitness costs. Therefore, we hypothesize that the re-emergence of MDR would require the H58 lineage to fall below 5% before recurrence of the resistance for safe reintroduction of the first-line drugs (Line 348-355).

Also, to mitigate this risk of recurrence, we emphasize on continuing clinical, laboratory, and genomic surveillance along with the introduction of typhoid conjugate vaccine (TCV). Monitoring of antimicrobial use and resistance will remain crucial for preventing the recurrence of MDR (Line 355-358).

Line 348-358: “However, reintroducing older drugs also risks the re-emergence of MDR with increased use. The initial H58 *Salmonella* Typhi isolates in the 1990s carried MDR genes on an IncHI1 plasmid. Recent H58 *Salmonella* Typhi isolates in Bangladesh and other endemic countries have integrated these MDR genes into a chromosomal island. This integration helps avoid plasmid-imposed fitness costs, which negatively impact bacterial reproduction and survival due to plasmid carriage [46, 56, 57]. Due to these genomic changes, the re-emergence of MDR would require the frequency of H58 lineage to fall below 5% before reintroduction of older drugs. Continuous clinical, laboratory, and genomic surveillance in the post-TCV era will be essential to secure a safe window for the use of the old antimicrobials [43]. Monitoring antimicrobial use and resistance remains crucial for successfully treating typhoid and other infections effectively.”

3. In line 96, the current treatments include azithromycin and third-generation cephalosporins like ceftriaxone. In Fig 4., you only showed a consumption pattern of azithromycin with a 38-fold increase in 24 years. What about the consumption pattern of ceftriaxone? Does it also have a significant increase in use?

- Thanks to the reviewer for their insightful comment regarding the consumption patterns of azithromycin and third-generation cephalosporins, specifically ceftriaxone, in our study.

We confirm that azithromycin and ceftriaxone are the preferred treatment for typhoid fever in Bangladesh. Unfortunately, we did not have access to the consumption data for ceftriaxone and thus, our analysis was limited only to azithromycin. We added this as a limitation of our study (Line 377-378).

However, our antimicrobial resistance (AMR) data showed a slight increase in the MIC for ceftriaxone, although it remains well below the resistance threshold (according to CLSI) (Line 312-315).

Line 377-378: “Additionally, antibiotic consumption data for amoxicillin, chloramphenicol, and ceftriaxone were unavailable.”

Line 312-315: “However, analysis of 35% (3,518/10,061) of the isolates detected between 1999 and 2019, demonstrated a gradual four-fold increase in ceftriaxone MIC to 0.12 mg/L. Although still below the non-susceptibility breakpoint of 1.0 mg/L [30]), we must remain vigilant and continue to carefully monitor the gradual increase in ceftriaxone MIC.”

**Reviewer #4:**

Minor Revision required.

- Thanks to the reviewer for their positive comments on our manuscript.

**Reviewer #5:**

NA

--------------------

**Summary and General Comments**

Use this section to provide overall comments, discuss strengths/weaknesses of the study, novelty, significance, general execution and scholarship. You may also include additional comments for the author, including concerns about dual publication, research ethics, or publication ethics. If requesting major revision, please articulate the new experiments that are needed.

**Reviewer #1:**

Over all Impression and General Comments:

The title "Trends in antimicrobial resistance amongst *Salmonella* Typhi in Bangladesh: a 24-year retrospective observational study (1999–2022)", effectively captures the essence of the study and provides a clear indication of its focus and scope. The study explores the changing patterns of antimicrobial resistance (AMR) in *Salmonella* Typhi bacteria in Bangladesh over the span of 24 years, from 1999 to 2022. Given the high burden of typhoid fever in Bangladesh and the global concern regarding antimicrobial resistance, this study would provide valuable insights into the dynamics of antibiotic resistance in a significant pathogen over an extended period. It would also inform strategies for combating antimicrobial resistance and improving the management of typhoid fever.

The methodology section provides a thorough description of the study's surveillance approach, laboratory procedures, antibiotic consumption analysis, and statistical methods. While the methodology demonstrates strengths in its comprehensive approach and standardized procedures, addressing the suggested areas for improvement would further enhance the clarity, transparency, and robustness of the methodology. The results section generally adheres to the analysis plan outlined in the methodology and effectively presents the main findings of the study.

The conclusions drawn in the study are well-supported by the data presented, and the limitations of the analysis are clearly described. The authors effectively discuss how the data contribute to advancing our understanding of the topic and address the public health relevance of the findings. Overall, the study provides valuable insights into antimicrobial resistance trends among Salmonella Typhi isolates and their implications for public health in Bangladesh. I recommend that authors consider the following minor suggestions for improvement of the manuscript.

- Thanks to the reviewer for their comments on the Methods section. We have addressed each comment below and made the respective improvements to the manuscript.

**Suggestions for Improvement:**

*Clarification of Sampling Strategy:* It would be beneficial to provide more clarity on the sampling strategy for blood cultures, particularly regarding the criteria for selecting cases for culture and potential biases introduced by physician discretion.

- Regarding the sampling strategy, we would like to clarify that our study did not include a calculated sample size. Instead, the data presented in our study is part of an ongoing laboratory surveillance started in 1999 and expanded across multiple microbiology labs at different hospital sites since 2004 (details of the surveillance are already provided in the Methods section; Line 124-139).

Our sampling strategy involved the inclusion of all blood culture-confirmed typhoid fever cases identified through this surveillance system. There were no criteria for selecting cases for blood culture except for the advice from attending physician. This approach aimed to capture a comprehensive and representative dataset of typhoid cases both in community and hospital settings. We acknowledge that physician discretion in advising blood cultures may introduce potential biases and we added it in the limitation section of the discussion (Line 367-370).

Line 367-370: “Fourth, the sampling strategy of our study did not include a calculated sample size, as it was conducted as part of an ongoing laboratory surveillance for enteric fever. Additionally, blood cultures were suggested by treating physicians, which may have introduced biases.”

*Transparency in MIC Testing:* While the methodology describes MIC testing for ceftriaxone and ciprofloxacin, it would be helpful to specify the criteria used to determine which isolates underwent MIC testing and any potential limitations associated with this approach.

- For MIC testing, we tested all isolates from 1999-2016 that were recoverable on plates for both ciprofloxacin and ceftriaxone. Additionally, a set of 299 isolates were randomly selected from 2017-2019, and tested for ceftriaxone MIC. We added these details in the Methods section of the manuscript (Lines 148-154).

Lines 148-154: “The minimum inhibitory concentrations (MICs) for ceftriaxone and ciprofloxacin were determined for two subsets of isolates (from 1999–2019 and 1999–2016 respectively) using the broth microdilution method (Sigma-Aldrich, MO, USA) [28, 29]. We determined MICs up to a maximum of 256 mg/L. All isolates collected before 2017 underwent MIC testing if available and recoverable on culture plates. Additionally, ceftriaxone MIC testing was performed on a random set of 299 isolates (considering hospital sites and year of isolation) collected between 2017 and 2019.”

*Justification for Exclusion of Outliers:* The rationale behind excluding specific ceftriaxone MIC outliers from the analysis should be explained in more detail to ensure transparency and justify the decision's impact on the interpretation of results.

- The decision to exclude the extremely high ceftriaxone MICs (MIC >256 mg/L) from all analyses was made because we could not measure the exact MICs of these two isolates. Our microdilution plate was designed to determine MIC values up to a maximum of 256 mg/L. We have included this in the Methods section (Line 148-151 and 159-161) and as a limitation in the Discussion section (Line 370-373).

Line 148-151: “The minimum inhibitory concentrations (MICs) for ceftriaxone and ciprofloxacin were determined for two subsets of isolates (from 1999–2019 and 1999–2016 respectively) using the broth microdilution method (Sigma-Aldrich, MO, USA) [28, 29]. We determined MICs up to a maximum of 256 mg/L.”

Line 159-161: “Ceftriaxone MIC of two resistant isolates (MIC >256 mg/L) were excluded from all related analyses, as the exact MICs of those isolates could not be determined.”

Line 370-373: “Fifth, our broth microdilution plates were designed to determine MICs up to a maximum of 256 mg/L, and it prevented us from accurately measuring the exact ceftriaxone MICs of the two resistant isolates, which were excluded from subsequent analysis.”

*Data Validation:* Given the reliance on commercial databases for antibiotic consumption data, it would be prudent to mention any measures taken to validate the accuracy and reliability of these data sources to enhance the credibility of the findings.

- We acknowledge that we could not find any other data source to validate the data points from commercial databases for antibiotic consumption data. We have expanded the limitations section to include this (Lines 373-378).

Line 373-378: “Finally, national antibiotic consumption data from IQVIA-MIDAS® database were used in this study, as regional or disease-specific data were unavailable. Since pharmaceutical sales data is proprietary information, we could not find any other source providing yearly comprehensive sales data of all antimicrobials available in Bangladesh. Consequently, we could not validate these data points with other sources. Additionally, antibiotic consumption data for amoxicillin, chloramphenicol, and ceftriaxone were unavailable.”

**Reviewer #2:**

The observational study describes Salmonella typhi antibiotic resistance in isolates from select health facilities in Dhaka, Bangladesh, over more than 20 years. The associations with use of those antibiotics in Bangladesh over the same period is explored. The authors suggest that the data supports the reintroduction of first-line drugs for empiric treatment of typhoid. The study and substantial data set (shared in supplementary material) provide a valuable resource for future studies - both as a baseline for further monitoring including evaluation of the impact of the roll-out of typhoid vaccines, and models to understand the development and transmission of AMR with antibiotic use/decline in use.

There are minor grammatical errors/typos (prominently the 2nd sentence of discussion) - indicating careful review the manuscript for these.

- We thank the reviewer for the comment. We have carefully gone through the manuscript again to take care of grammatical errors/typos.

The 2^nd^ sentence of the discussion:

Line 282-284: In resource-limited endemic countries like Bangladesh, a large proportion of typhoid fever cases is largely treated in the community, and more severe cases are treated in the hospital.”

In the methods section it is stated that, “All isolates collected before 2017 underwent MIC testing if recoverable on plates.” Please indicate in the results what proportion of isolates were recoverable, and in the discussion comment on potential bias in selection of isolates for MIC based on recoverability, if this is a potential weakness.

- For MIC testing, we could recover 61% of the isolates before 2017. We added the numbers in Line 216-220. We also revised a sentence to comment on this in the discussion (Line 309-313).

Line 216-220: “Non-susceptibility to ciprofloxacin in *Salmonella* Typhi was notably high, reaching 95% in 2001 from 56% in 1999, and remained above 90% throughout the study period. In 2022, 98% (1,266/1,298) of the isolates were non-susceptible to ciprofloxacin (Fig 2A). We further examined the non-susceptibility trend to ciprofloxacin using MIC data from 3,214 isolates recovered from 5,289 pre-2017 isolates (61%; Fig 3A).”

Line 309-313: “The current empirical typhoid treatment in the country is either ceftriaxone or oral azithromycin. Only two ceftriaxone-resistant *Salmonella* Typhi isolates were identified in our study (in 1999 and 2000). However, analysis of 35% (3,518/10,061) of the isolates detected between 1999 and 2019, demonstrated a gradual four-fold increase in ceftriaxone MIC to 0.12 mg/L.”

While the study and its findings very useful, and the limitations are discussed, I feel the manuscript would be improved by elaboration on specific issues, and richer discussion/contextualisation of others. These are related to the causal relationship between antibiotic use and antimicrobial resistance, and the implications thereof for reintroduction of first-line drugs, and are alluded to in the sections below.

- We thank the reviewer for the comments and have made changes to the manuscript to address them.

Why are the antibiotic consumption patterns of chloramphenicol and amoxicillin not reported? When did their use for typhoid decline? When did the empirical typhoid treatment in Bangladesh change to cefriaxone and azithromycin?

- Although we analyzed the antibiotic consumption data for cotrimoxazole, ciprofloxacin, and azithromycin, data for amoxicillin, chloramphenicol, and ceftriaxone were unavailable. We have included this statement as a limitation of our study (Lines 377-378).

However, use of amoxicillin, cotrimoxazole, and chloramphenicol for typhoid treatment began to decline in the 2000s and was replaced by ciprofloxacin. In 2011, based on recommendations from the WHO, ceftriaxone and azithromycin have been used for typhoid treatment, replacing ciprofloxacin. We have included this information in the Introduction (Lines 92-99).

Lines 92-99: “Management of typhoid fever is becoming increasingly difficult with rising AMR. Multi-drug resistant (MDR, defined as the concurrent resistance to amoxicillin, chloramphenicol, and co-trimoxazole) *Salmonella* Typhi emerged in the 1970s [12, 13]. This led to ciprofloxacin as the primary treatment choice by the mid-1980s, but widespread resistance followed. By the mid-2010s, over 90% of *Salmonella* Typhi in South Asia exhibited non-susceptibility to ciprofloxacin [14]. The current treatment options include azithromycin and third-generation cephalosporins such as ceftriaxone [13], but there are increasing reports of resistance to both the drugs [15-20].”

Line 377-378: “Additionally, antibiotic consumption data for amoxicillin, chloramphenicol, and ceftriaxone were unavailable.”

What portion of the consumption of an antibiotic is likely attributed to the indication of typhoid? Does antibiotic use for other indications (in humans and agriculture) contribute to S typhi resistance in Bangladesh? Is anything known of this? Is it likely their continued use for other indications that sustains resistance just under 20% for the first-line antibiotics?

- While we know that typhoid contributes significantly to human antibiotic consumption in Bangladesh due to its high incidence, we do not have data regarding the proportion of antibiotic consumption specifically due to typhoid. Currently, azithromycin and ceftriaxone are used for typhoid treatment. It is possible to understand the indications (human or agriculture) for which an antibiotic is used in Bangladesh; however, this information is proprietary, and we do not have access to it. It is likely that antibiotic use for other indications is one of the reasons why resistance to first-line antibiotics has remained just under 20%. Other factors, including environmental fitness, phage resistance, and virulence, may also play a role in the observed distribution of circulating *Salmonella* Typhi strains in the country. We thank the reviewer for these comments, as they provide areas for future investigation.

What factors/variables should future antibiotic-use databases consider including to support evaluation of the association between antibiotic use and AMR, including S typhi antibiotic resistance?

- Thank you for this comment. All the data used in this manuscript came from the IQVIA-MIDAS® database. We were not able to extract antibiotic use indications (for typhoid or other indications) or obtain information for amoxicillin, chloramphenicol, and ceftriaxone from this database. If antibiotic-use databases could provide information regarding the specific indications for prescriptions, it would be very beneficial in establishing a link between antibiotic consumption and *Salmonella* Typhi AMR. However, such recommendations are beyond the scope of this present study.

Is there relevant precedent for reverting to empiric treatment of typhoid with first-line drugs with historical resistance? How low should the % resistance be before reintroduction for the indication? Is it useful to consider the molecular mechanisms of resistance when considering the robustness on reintroduction of a drug with historical resistance? Would genomic evaluation of isolates also be useful in this regard? Would additional surveillance to that already in place be necessary to monitor for re-emergence of resistance - what monitoring should be recommended?

- We thank the reviewer for this comment. In Samoa Island, practitioners continue using amoxicillin as a primary treatment as they have not encountered MDR issues due to a locally circulating non-MDR strains. According to the WHO guidelines, the resistance should fall below 5% before reintroduction can be supported. The molecular mechanism of resistance to the first line of drugs is well-known in *Salmonella* Typhi. Genomic evaluation will play a crucial role if re-introduction is considered. Our organization and others in Bangladesh already have the genomic surveillance in place to track resistance in *Salmonella* Typhi, if re-introduction is considered. We mention these monitoring systems in the Discussion section, lines 348-358.

Lines 348-358: “However, reintroducing older drugs also risks the re-emergence of MDR with increased use. The initial H58 *Salmonella* Typhi isolates in the 1990s carried MDR genes on an IncHI1 plasmid. Recent H58 *Salmonella* Typhi isolates in Bangladesh and other endemic countries have integrated these MDR genes into a chromosomal island. This integration helps avoid plasmid-imposed fitness costs, which negatively impact bacterial reproduction and survival due to plasmid carriage [46, 56, 57]. Due to these genomic changes, the re-emergence of MDR would require the frequency of H58 lineage to fall below 5% before reintroduction of older drugs. Continuous clinical, laboratory, and genomic surveillance in the post-TCV era will be essential to secure a safe window for the use of the old antimicrobials [43]. Monitoring antimicrobial use and resistance remains crucial for successfully treating typhoid and other infections effectively.”

The “MIC creep” within the susceptible range for ceftriaxone is interesting. Is this expected with appropriate use of good quality antibiotic products or does it indicate a need to explore inadequate treatment due to health care practitioner, or patient factors, or are there concerns about formulation quality. It also raises the issue of whether the PK/PD (pharmacokinetic/pharmacodynamic) targets are well defined and whether dosing practices readily achieve the target/s.

- We thank the reviewer for the comment and agree that appropriate use of good quality antibiotic products would have likely mitigated some of these effects. The “MIC creep” we observe is likely due to several factors, as the reviewer mentioned. Typhoid treatment in Bangladesh is often done empirically, with different third-generation cephalosporin products from several local pharmaceutical companies using various formulation strategies. Furthermore, typhoid treatment occurs in diverse healthcare settings (hospitals, clinics, pharmacies) with different prescribing practices. All these factors are likely contributing to the observed increase in MICs. We have added a sentence in the discussion to comment on this (Lines 314-317).

Line 314-317: “Although still below the non-susceptibility breakpoint of 1.0 mg/L [30]), we must remain vigilant and continue to carefully monitor the gradual increase in ceftriaxone MIC. In addition, consumption of this drug must also be carefully controlled, considering the narrowing window of treatment for typhoid.”

**Reviewer #3:**

Authors examined the trends in antimicrobial resistance in Salmonella Typhi isolates collected from Bangladesh and compared them with antibiotic consumption data. They conducted typhoid fever surveillance from 1999 to 2022, collecting data from two largest pediatric hospitals in Dhaka and three community-based clinics. The decline in resistance to first-line drugs and multidrug resistance was observed over the 24 years. Resistance to cotrimoxazole went down (80% in 2002 to 20% in 2022) as consumption of cotrimoxazole decreased. Ciprofloxacin non-susceptibility remained high (56% in 1999 to 98% in 2022) with unchanged consumption. Ceftriaxone resistance was low but showed an increase in minimum inhibitory concentration. Azithromycin consumption increased, but resistance remained low. Authors suggest that reintroducing first-line antimicrobials could be an empirical treatment option for typhoid fever in Bangladesh and provide a baseline for monitoring future interventions on typhoid burden and AMR.

It is well-written and of significance in designing future intervention strategies to lessen the typhoid burden in Bangladesh.

Thanks to the reviewer for their positive feedback on our manuscript.

**Reviewer #4:**

(No Response)

**Reviewer #5:**

Tanmoy and colleagues conducted a comprehensive investigation into antimicrobial resistance trends in Salmonella Typhi isolates from Bangladesh spanning 24 years (1999–2022). The study compares these trends with antibiotic consumption data to inform effective antibiotic stewardship strategies. Notably, the results reveal a significant decline in resistance to first-line drugs (amoxicillin, chloramphenicol, and cotrimoxazole) and multidrug resistance over the surveillance period, potentially attributed to the reduction in antibiotic consumption. This is an interesting manuscript.

Thanks to the reviewer for their positive feedback on our manuscript.
