# Supplementary material for: Trends in antimicrobial resistance amongst Salmonella Typhi in Bangladesh: A 24-year retrospective observational study (1999–2022)
Source: PLoS Negl Trop Dis. 2024 Oct 4;18(10):e0012558. doi: 10.1371/journal.pntd.0012558 (PMC11482714; doi:10.1371/journal.pntd.0012558)
Supplement: Attachment — Submitted filename: Response_to_Reviewer_Comments_Revision2.docx [file pntd.0012558.s004.docx]

**Reviewer's Responses to Questions**

Key Review Criteria Required for Acceptance?

As you describe the new analyses required for acceptance, please consider the following:

**Methods**

-Are the objectives of the study clearly articulated with a clear testable hypothesis stated?

-Is the study design appropriate to address the stated objectives?

-Is the population clearly described and appropriate for the hypothesis being tested?

-Is the sample size sufficient to ensure adequate power to address the hypothesis being tested?

-Were correct statistical analysis used to support conclusions?

-Are there concerns about ethical or regulatory requirements being met?

**Reviewer #1:** The current study method is robust and I recommend it be accepted

We thank the reviewer for the positive feedback.

**Reviewer #3:** The methods are clear and well-written.

Thanks to the reviewer for their positive feedback.

**Reviewer #5:** NA

--------------------

**Results**

-Does the analysis presented match the analysis plan?

-Are the results clearly and completely presented?

-Are the figures (Tables, Images) of sufficient quality for clarity?

**Reviewer #1:** The results section is well-structured and provides clear findings from the study. Below is a review with comments on clarity, completeness, and potential areas for improvement:

1. Include exact years for "recent years" to avoid ambiguity.

2. Ensure clarity on the exact number of MDR cases per year for better understanding.

3. On drug resistance:

-Ciprofloxacin: Clarify the significance of stable MIC trends in the context of clinical outcomes.

-Ceftriaxone: Discuss the clinical relevance of the four-fold MIC increase despite remaining below the susceptibility breakpoint.

-Azithromycin: Specify the potential implications of these resistance rates on treatment guidelines.

4. Correlation Between AMR and Antimicrobial Consumption: Include a brief discussion on the lack of significant correlation for ciprofloxacin to provide context

We appreciate the reviewer for the positive feedback. We revised the manuscript based on your suggestions. Please check the individual responses in the Summary and General Comments section below.

**Reviewer #3:** The results are clear and well presented in the figures.

Thanks to the reviewer for their positive comments.

**Reviewer #5:** NA

--------------------

**Conclusions**

-Are the conclusions supported by the data presented?

-Are the limitations of analysis clearly described?

-Do the authors discuss how these data can be helpful to advance our understanding of the topic under study?

-Is public health relevance addressed?

**Reviewer #1:** The conclusion is reasonably supported by the data.

The limitations of the study are not explicitly described in the conclusion section provided.

Recommendation: The authors should include a discussion of limitations, such as potential biases in blood culture practices, variability in sample collection, or changes in diagnostic technologies over time.

Thanks to the reviewer for the comment. We have included a discussion on the limitations in the Discussion section (lines 381-399). For the three potential biases the reviewer has included:

1. Blood culture training and practices remained consistent throughout the study.
2. Variability in sample collection should not affect the AMR pattern of *Salmonella* Typhi strains that we describe.
3. Changes in diagnostic technologies have occurred (including the use of BACTEC since 2016 in the laboratory). We have added the following lines in the Methods section (145-153):

“In short, blood samples were sent to the microbiology laboratories of all the study hospitals where samples were cultured on blood, chocolate, and MacConkey agar plates. From 2016, blood specimens were incubated in BACTEC culture systems for up to 72 hours. BEEP-positive samples were then cultured on blood, chocolate, and MacConkey agar plates. Upon growth, biochemical (Klingler’s Iron agar, Simmons citrate agar, motility-indole, and urea agar tests) and slide-agglutination tests (using *Salmonella* agglutinating antisera; Thermo Scientific, MA, USA) were done for identification of *Salmonella* Typhi.”

However, this change will not affect the AMR patterns described in the study and hence was not included in the Discussion limitations section.

**Reviewer #3:** The conclusions are appropriate, and limitation was clearly described.

Thanks to the reviewer for their positive comments.

**Reviewer #5:** NA

-------------------

**Editorial and Data Presentation Modifications?**

Use this section for editorial suggestions as well as relatively minor modifications of existing data that would enhance clarity. If the only modifications needed are minor and/or editorial, you may wish to recommend “Minor Revision” or “Accept”.

**Reviewer #1:** Minor revision

We thank the reviewer for their positive insights on our manuscript.

**Reviewer #3:** I would recommend accepting this manuscript for publication.

We appreciate the reviewer’s positive decision for our manuscript.

**Reviewer #5:** NA

--------------------

**Summary and General Comments**

Use this section to provide overall comments, discuss strengths/weaknesses of the study, novelty, significance, general execution and scholarship. You may also include additional comments for the author, including concerns about dual publication, research ethics, or publication ethics. If requesting major revision, please articulate the new experiments that are needed.

**Reviewer #1:** General Impression:

The abstract presents a clear objective for the study, which is to explore AMR trends of *Salmonella* Typhi isolates in Bangladesh and compare them with antibiotic consumption to optimize antibiotic stewardship strategies. However, the abstract does not explicitly state a testable hypothesis. For a more complete and scientifically rigorous abstract, it would be beneficial to articulate a clear hypothesis. Including a testable hypothesis can provide a stronger foundation for the study. For example, Authors might hypothesize that "declining antibiotic consumption is associated with decreased AMR in Salmonella Typhi in Bangladesh."

We thank the reviewer for this comment. However, this is an observational study aimed at devising a hypothesis, rather than testing one. Hence, the addition of this section is not required for this abstract.

Methods section: The study design involves long-term surveillance of typhoid fever, which is appropriate for understanding AMR trends over time. The use of multiple hospitals and clinics provides a comprehensive view of the situation in Dhaka, Bangladesh.

The use of blood cultures and standard microbiological techniques to identify and test Salmonella Typhi is appropriate for this type of study. The statistical methods are clearly described, and the use of software (Stata and R) is mentioned, ensuring reproducibility.

The results section is well-structured and provides clear findings from the study. The trends for amoxicillin, chloramphenicol, and cotrimoxazole are clearly described. The percentage values and time points (e.g., 2002 peak, recent years) are provided. The trend of MDR decline is well-documented, and the average percentage and confidence intervals are provided.

Ethical approval was obtained from the relevant Ethics Review Committees. Informed consent procedures are described for hospitalized cases, and the retrospective inclusion of outpatient data without identifiable information is noted. This meets standard ethical and regulatory requirements.

Minor Edits for Improvement:

We appreciate the reviewer’s insightful comments and suggestions. We believe they have enhanced the clarity and quality of our manuscript. Please see our responses to the individual comments below.

1. Include a statement of the objectives and hypothesis in the introduction or background section.

We thank the reviewer for this comment. However, this is observational study aimed at devising a hypothesis, rather than testing one. Hence, the addition of hypothesis in this section is not required for this Introduction/background section. We have listed the objectives of the study in Lines 119-121.

“In this study, we aim to elucidate correlations between AMR and antibiotic consumption, providing a baseline to guide the design of antibiotic stewardship policies.”

2. Ensure all technical terms and abbreviations (e.g., DDD) are defined when first mentioned

Thanks to the reviewer for pointing it out. DDD is already elaborated in the main text. We added the elaboration in the abstract as well (Line 41).

3. Include exact years for "recent years" to avoid ambiguity.

We revised the sentences and added the exact years in Line 211.

“In 2002, resistance peaked at 80%, but decreased to less than 20% in recent years (since 2020) for all three first-line antimicrobials (Fig 1A-C).”

4. Ensure clarity on the exact number of MDR cases per year for better understanding.

Results of MDR cases over the years are given in Lines 210-214, with average percentage, range (with confidence interval) of resistance cases.

“In 2002, resistance peaked at 80%, but decreased to less than 20% in recent years (since 2020) for all three first-line antimicrobials (Fig 1A-C). This decline was also reflected in multidrug resistance (MDR), which was 17% in 2022 (Fig 1D). The percentage of MDR isolates remained ≤26% since 2010 (average 20%; 18% – 23% at 95% Confidence Interval, CI) and ≤19% since 2017 (average 17%; 15%–19% at 95% CI).”

More details have been illustrated in Figure 1D.

5. On drug resistance:

-Ciprofloxacin: Clarify the significance of stable MIC trends in the context of clinical outcomes.

We discussed the clinical significance of the stable MIC pattern of ciprofloxacin in Lines 315-323.

“Both ciprofloxacin non-susceptibility and its consumption, however, remained high and unchanged throughout the study period, indicating its ongoing ineffectiveness for treating typhoid in Bangladesh. This stable resistance pattern over 24 years also suggests that changes in clinical outcomes due to ciprofloxacin use are unlikely in the near future.”

-Ceftriaxone: Discuss the clinical relevance of the four-fold MIC increase despite remaining below the susceptibility breakpoint.

We addressed the clinical relevance of the four-fold MIC increase for ceftriaxone in Lines 325-332.

“The current empirical typhoid treatment in the country is either ceftriaxone or oral azithromycin. Only two ceftriaxone-resistant *Salmonella* Typhi isolates were identified in our study (in 1999 and 2000). However, analysis of 35% (3,518/10,061) of the isolates detected between 1999 and 2019, demonstrated a gradual four-fold increase in ceftriaxone MIC to 0.12 mg/L. Although still below the non-susceptibility breakpoint of 1.0 mg/L [30] and not clinically threatening yet, we must remain vigilant and continue to carefully monitor the gradual increase in ceftriaxone MIC. Thus, clinical consumption of this drug must also be carefully controlled, considering the narrowing window of treatment for typhoid.”

-Azithromycin: Specify the potential implications of these resistance rates on treatment guidelines.

We highlighted the implications of azithromycin resistance rates on typhoid treatment in Lines 343-348.

“However, our study does not demonstrate any increasing trend of azithromycin resistance. This sporadic resistance pattern could impact treatment guidelines, as azithromycin is a primary treating option for typhoid fever in Bangladesh. Spread of this resistance could limit its effectiveness and necessitate re-evaluating azithromycin use. Continuous monitoring is essential to ensure its efficacy and update guidelines to avoid compromising patient outcomes.”

6. Correlation Between AMR and Antimicrobial Consumption: Include a brief discussion on the lack of significant correlation for ciprofloxacin to provide context

We discussed the lack of significant correlation between ciprofloxacin non-susceptibility and its use in Lines 315-323.

“In addition, the lack of significant correlation between ciprofloxacin non-susceptibility and its use (r = 0.21; p = 0.38 at 95% CI) suggests the involvement of other factors, possibly genomic changes. Unlike MDR, ciprofloxacin non-susceptibility in *Salmonella* Typhi is generally driven by mutations in the *gyrA* and *parC* genes, which exert no fitness cost and are naturally maintained even if ciprofloxacin use is reduced [44].”

**Reviewer #3:** Reviewers' suggestions were well addressed.

We appreciate the positive response from the reviewer.

**Reviewer #5:** I am satisfied with the revised version and fully support its publication.

We thank the reviewer for their positive comment.

--------------------

PLOS authors have the option to publish the peer review history of their article (<a href="<https://journals.plos.org/plosntds/s/editorial-and-peer-review-process#loc-peer-review-history>" target="_blank">what does this mean?</a>). If published, this will include your full peer review and any attached files.

If you choose “no”, your identity will remain anonymous but your review may still be made public.

Do you want your identity to be public for this peer review?</b> For information about this choice, including consent withdrawal, please see our <a href="<https://www.plos.org/privacy-policy>" target="_blank">Privacy Policy</a>.

**Reviewer #1:** No

**Reviewer #3:** No

**Reviewer #5:** No
